# Enhancing soccer goalkeepers penalty dive kinematics with instructional video and laterality insights in field conditions

**DOI:** 10.1038/s41598-024-60074-x

**Published:** 2024-05-03

**Authors:** Rafael Luiz Martins Monteiro, Carlos Cesar Arruda dos Santos, Patrick Blauberger, Daniel Link, Tiago Guedes Russomanno, Ariany Klein Tahara, Abel Gonçalves Chinaglia, Paulo Roberto Pereira Santiago

**Affiliations:** 1 Biomechanics and Motor Control Laboratory, Ribeirão Preto Medical School, University of São Paulo, Av. Bandeirantes, 3900, Monte Alegre, Ribeirão Preto, SP 14049-900 Brazil; 2 School of Physical Education and Sports of Ribeirão Preto, University of São Paulo, Ribeirão Preto, 14040-907 Brazil; 3 Chair of Performance Analysis and Sports Informatics, Technical University of Munich, 80992 Munich, Germany

**Keywords:** Markerless, 3D reconstruction, Biomechanics, OpenPose, Football, Musculoskeletal system, Biomedical engineering, Computer science, Time series, Techniques and instrumentation, Imaging techniques, Data processing, Motor control

## Abstract

This study aimed to analyze the effect of laterality and instructional video on the soccer goalkeepers’ dive kinematics in penalty. Eight goalkeepers from youth categories (U15, U17, U20) were randomly divided into control (CG) and video instruction groups (VG). The latter performed 20 penalty defense trials on the field with balls launched by a machine, ten before and after watching a video instruction to improve the diving kinematics. The CG only performed the dives. Three cameras recorded the collections. A markerless motion capture technique (OpenPose) was used for identification and tracking of joints and anatomical references on video. The pose data were used for 3D reconstruction. In the post-instruction situation, the VG presented differences in comparison to the CG in the: knee flexion/extension angle, time to reach peak resultant velocity, frontal step distance, and frontal departure angle, which generated greater acceleration during the dive. Non-dominant leg side dives had higher resultant velocity during 88.4 – 100% of the diving cycle, different knee flexion/extension angle, and higher values ​​in the frontal step distance. The instructional video generated an acute change in the diving movement pattern of young goalkeepers when comparing the control and the video instruction group in the post condition.

## Introduction

The penalty kick is the set play with the highest probability of scoring a goal^1,2^ as it is a duel only between the penalty kicker and the goalkeeper. Some people say it is a lottery, but science has proven that it is not. The goalkeeper and the penalty taker have factors that influences their performances and strategies that they can benefit from. A revision has pointed out important factors that may influence the players: coping with stressful situations, non-verbal behavior, motivational fit and anxiety^3^. Other factors were also highlighted by different studies: athlete's reputation^4^, positioning^5,6^, uniform color^7,8^, goalkeeper and penalty taker strategies^9–11^ and penalty kick direction^3,12^^.^ All these factors influence the main actions in the penalty, the kick and the diving save.

The penalty kick kinematics has been studied in many ways, exploring the support leg^13,14^, kicking techniques^15,16^, approach angle^17^, velocity and accuracy in different categories^18^ and other factors^19^. But only few studies have focused on the goalkeepers’ diving save kinematics. Research with elite goalkeepers concluded that the lower limb contralateral to the diving side has a greater contribution to the goalkeeper center of mass (CM) velocity and specifically the contralateral hip extensors^20–22^. They also did not find differences in performance between the diving sides. Other studies found some laterality effects on the diving kinematics. Lower CM velocity in the ball contact moment was found in dives to the non-dominant lower limb (NDLL) side in elite goalkeepers^23^. Greater horizontal and resultant CM displacement was found in dives for the NDLL side in amateur and professional goalkeepers^24^. Other attested that the dives to the NDLL side have greater variability between the performance of consecutive dives in relation to the dominant lower limb (DLL) side but this research evaluated only 1 goalkeeper^25^.

However, all these diving kinematic researches were carried out in laboratories and only 1 without stationary balls^21^, therefore, field studies are necessary to determine whether the findings are applicable in situations more representative of actual game conditions. The use of markerless methods for kinematic analysis can make it possible. An example is the OpenPose, a human pose detection library that allows the identification of joints and anatomical points in videos through skeleton detection algorithms, providing an accessible and reproducible way for kinematic analysis^26–29^. The use of this type of resource allows data collections in the field and without markers, which is promising and innovative as it allows tasks to be done in the most natural way possible, giving greater ecological validity to the experiment.

The methods to improve the goalkeeper's kinematic dive in penalty are still not clear in the literature. A study applied a combined technical and physical 12-week training program that improved the CM horizontal velocity and power at the contralateral push-off in the penalty, increased the push-off feet preparatory stance width and decreased the diving time. The technical training involved the distance between the feet in the dive preparatory posture adjustment to 75% of the leg length using a personalized stick for the goalkeepers’ feet positioning^22^. The scientific literature lacks studies that propose and evaluate methods to improve the goalkeepers' kinematics in the diving save.

Regarding motor skills acquisition and improvement in sports the kind of instruction and how it is passed can lead to different results. Several research has investigated the use of video instructions on sports. Studies using instructions of internal (i.e., own body) and external (i.e., body effect on the environment) attentional focus compared oral and video instructions and showed that the second is as efficient or better in many sport tasks, as: throwing^30^, golf ^31^, discus, hamme^32^, tennis^33^ and landing tasks^34^. Some authors also suggested the creation of specific audiovisual materials for learning different motor skills^34^. However, despite the various evidence regarding the benefit of video instructions the literature still lacks studies that analyze the effect of an instructional video on soccer goalkeeper diving  kinematics.

Therefore, the present study aimed to analyze the effect of laterality and video instruction in the diving kinematics of soccer goalkeepers in penalty. The initial hypothesis was that after watching a diving save video instruction the goalkeepers would improve diving performance which would appear in the diving kinematics variables analyzed, and that the dives to the NDLL side would present better diving performance when compared with the DLL side trials.

## Methods

### Participants

Eight soccer goalkeepers from the youth categories (U15, U17 and U20) of a club that plays at the A2 series of the São Paulo state championship participated in this study. Participants were randomly divided into a control group (CG) (n = 4; age = 16.9 ± 2.4 years old; mass = 75.9 ± 10.4 kg; height = 1.77 ± 0.09 m; training experience in the position = 6.8 ± 1.9 years; training routine = 5 ± 2 days a week) and video group (VG) (n = 4; age = 16.4 ± 2 years; mass = 84.5 ± 11.2 kg; height = 1.88 ± 0.09 m; training experience in the position = 5.8 ± 4.9 years, training routine = 5 ± 2 days a week). Both groups had 3 right-footed players and 1 left-footed.

The School of Physical Education and Sport of Ribeirão Preto Ethics Committee approved all the experimental procedures (CAAE: 24,268,719.0.0000.5659). The experiments were conducted in accordance with established ethical standards, as outlined in Resolution 466/12 of the National Health Council of Brazil dated December 12, 2012 (BRASIL, 2012), and the Resolution of Helsinki (2001). Written informed consent was obtained from the participants and their legal guardian(s).

### Instruments

**Figure 1:**
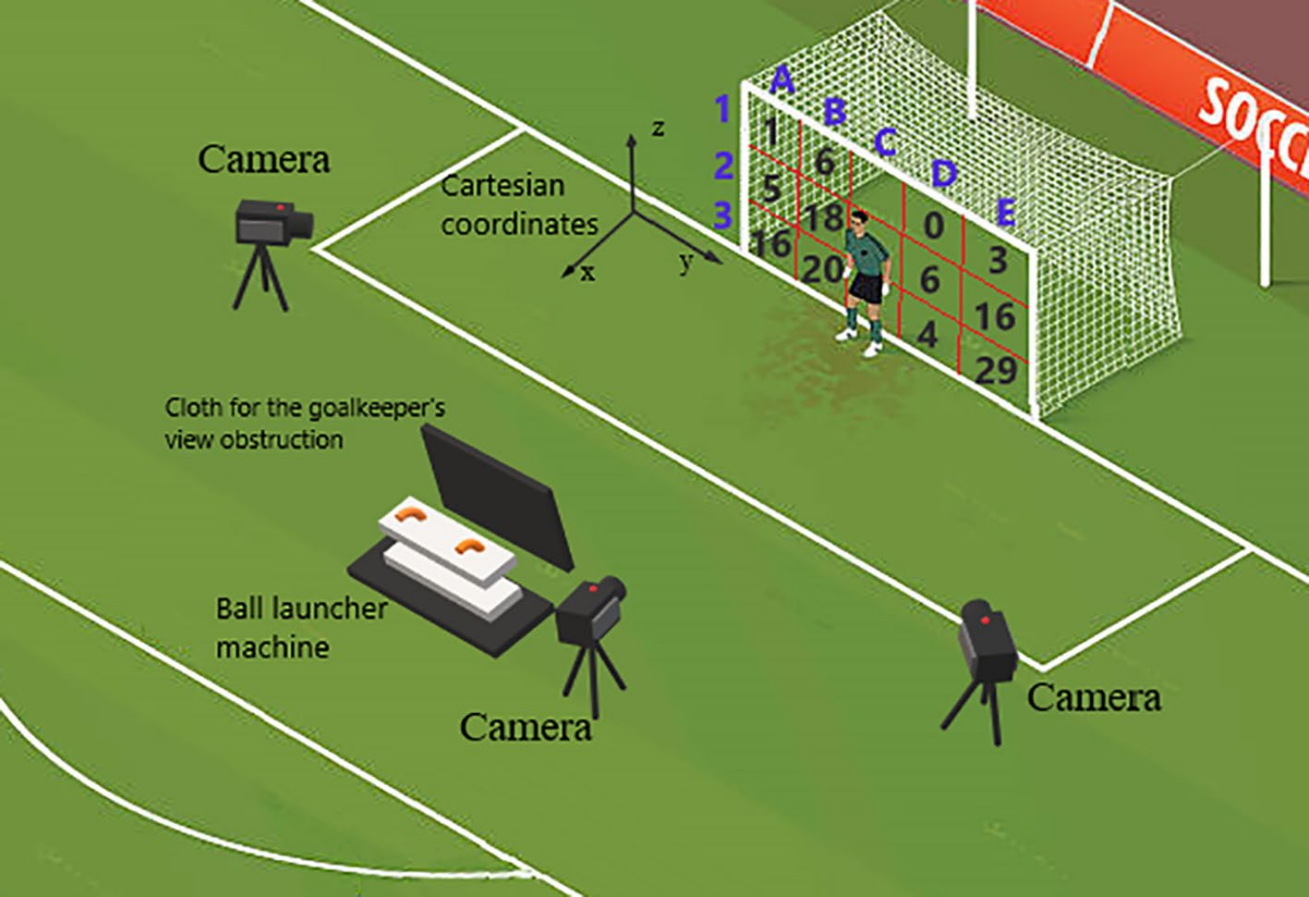
The data collection experimental setup. References of the camera positioning, ball launcher machine, cloth for goalkeeper's view obstruction and cartesian coordinates. Goal divided into 15 quadrants with the number of balls launched in each one.

For data collection, 3 cameras (GoPro HERO 3 + Black Edition) were set at an acquisition frequency of 120 Hz. A camera was placed in front of the goal and the other 2 arranged on different sides with a diagonal view, all pointing towards the center of the goal (Fig. 1). In order to identify the volunteer's lateral preference, the Global Lateral Preference Inventory (IPLAG)^35^ was used. A notebook played the instructional video for the goalkeepers.

For the soccer kick representation, a soccer ball launcher machine was placed in the penalty mark and a cloth to obstruct the goalkeeper's view of the side where the ball would be thrown were used^36^ (Fig. 1). This machine was used to standardize the ball launch location and speed (64.7 ± 5 km/h)^25^. Official size balls proposed by the Brazilian Football Confederation^37^, with diameter between 68 and 70 cm were used.

### Instructional video

A 6 min and 20 s video containing instructions to improve the diving movement pattern of soccer goalkeepers at the penalty was elaborated in an oral Power Point format presentation with language accessible to goalkeepers. The kind of instructions used were with internal and external focus and by analogy^38–40^. The instructions were written on the slides and accompanied by images, videos, and oral explanation. At the end of the video, a summary was made so that the goalkeepers could remember all the instructions passed. To prepare the instructional video, references from the literature that explored the goalkeeper’s diving kinematics on penalty kicks and stretching-shortening cycles of the muscles were used^20,21,23,25,36,41–43^, in addition to consultation with biomechanics specialists and goalkeeping coaches.

The tips in the instructional video were: (1) on the support base for the dive keep the feet apart at a distance corresponding to 75% of the leg length (this value was calculated for each VG athlete and they were placed in the suggested position for them to get used to it)^43^; (2) use the frontal step with the leg ipsilateral to the dive side at the impulsion moment^24^; (3) seek to reach the highest speed in the shortest possible time^20,21,36,43^; (4) use the muscle elastic component (rapid stretch–shortening cycle) and the arms on the impulse^41,42^; (5) dive with a frontal departure angle of 18° (to cover the smallest distance between the center of the goal and the ball trajectory to the goal corners).

### Experimental procedures

The data was collected at the goalkeepers’ training site. Initially the cameras, ball launcher machine and cloth for goalkeeper’s vision obstruction was positioned in a standardized setup (Fig. 1). In sequence, the volunteers answered to the IPLAG to identify the lower limbs lateral preference. Before starting the dives execution, the goalkeepers performed a 5–10 min warm-up structured for the muscles to reach an optimal state for the collection task demand^44^. The final part of the warm-up was the execution of 2 jumps to the right and 2 to the left, to familiarize the goalkeeper with the penalty save dynamics with balls launched. For data collection, the goalkeeper was instructed to stay at the center of the goal, facing away from the penalty mark while the ball launcher machine was directed to the side the goalkeeper should jump. Then the goalkeepers were instructed to turn facing the penalty mark and advised that the collection had started and the ball would be launched.

Participants were instructed to perform the dive with maximum impulsion regardless of where the ball is released and were advised that the number of defenses made was not the focus of the study. The trials were only validated when the goalkeepers dived to the correct side in which the ball was launched. The final distribution of the balls launch sites in the validated dives are presented in Fig. 1. It is worth mentioning that several dives in region B and D were discarded because the ball launch harmed the goalkeepers’ impulsion biomechanics. Those who were kept had the balls thrown close to quadrants A and E. In relation to quadrant 1, there were also dives discarded when goalkeepers showed a vertical movement pattern inadequate for the analysis pretended and those who were kept had the ball thrown near quadrant 2. The final height that the balls were launched was close to a normal penalty distribution being respectively: lower third 55.64% and 56.6%, middle 36.29% and 30.4%, and upper third 8.06% and 12.9% (Fig. 1)^9^.

The VG performed a total of 20 dives, 10 before (VGPRE) and 10 after the video instruction (VGPOST). Totalizing 10 right dives, 5 before and 5 after the instructional video and 10 for the left side with the same number of trials in each condition. The diving execution side order was chosen randomly to not influence the results. The recovery time between attempts was equal or greater than 90 s. During the instructional video execution, VG participants were able to clear any doubts with the researchers and they could perform 2 dives to each side for the instructions given adaptation. The CG performed the same procedures as the VG, however, at the moment that the instructional video would be shown the volunteers had a rest period equivalent to the video's runtime, without receiving any kind of instruction or feedback. In other words, they performed the 10 initial dives (CGPRE), rested, and performed another block of 10 trials (CGPOST).

### Data processing

The lower limbs lateral preference was collected by IPLAG. It provides a numerical scale in which: 1 = strongly left-footed; 2 = moderate left-footed; 3 = ambidextrous or without preference; 4 = moderate right-footed; 5 = strongly right-footed. However, to separate the trials between the DLL side and NDLL side the volunteers classified as 1 and 2 were grouped in left-footed and the ones classified as 4 and 5 formed the right-footed group. No one was classified in category 3.

**Figure 2:**
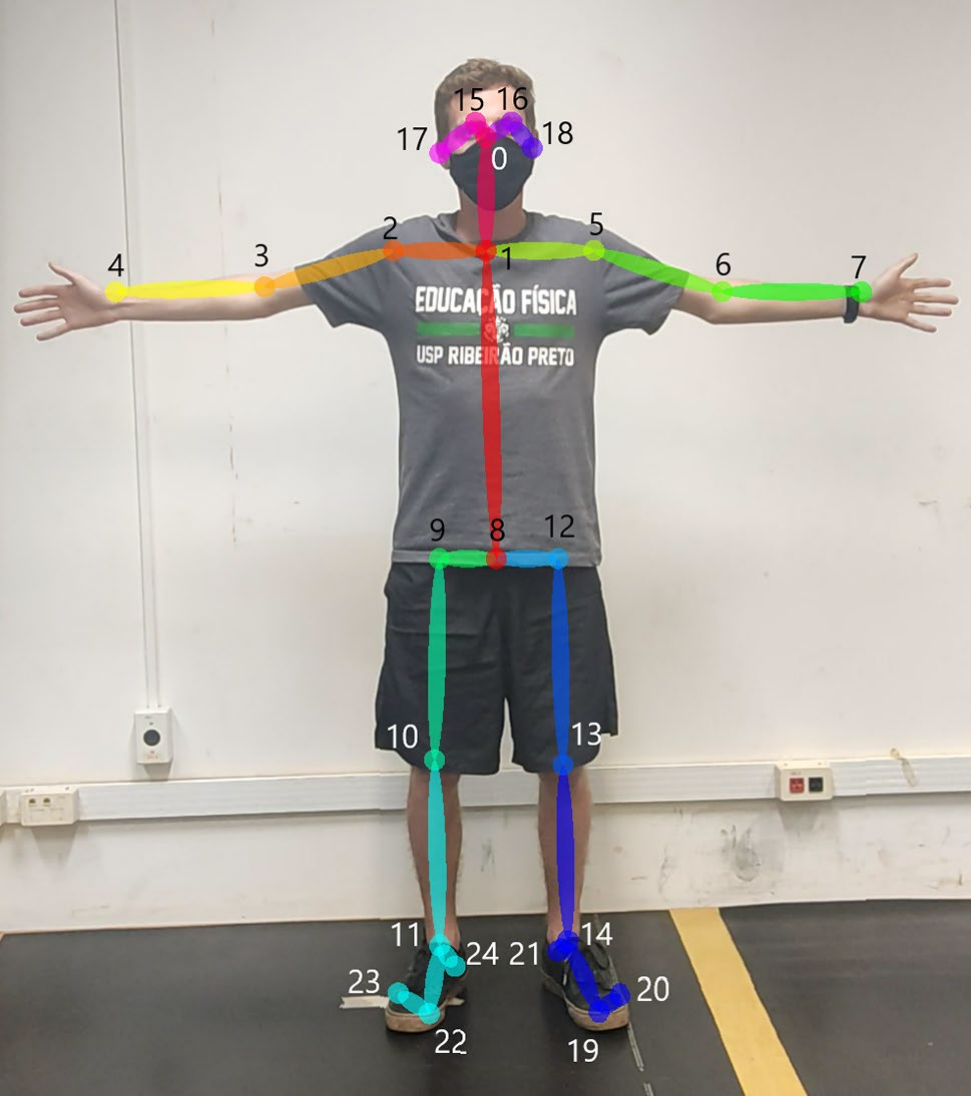
Identification of joints and anatomical points by OpenPose.

For kinematic analysis, the OpenPose, was used. It allows the identification of joints and anatomical points in videos through skeleton detection algorithms (Fig. 2). All data were reviewed and when the 2D coordinate obtained by OpenPose was missing from the goalkeeper's body it was manually corrected in the software Dvideow (v. 1.0.0.1)^45^. Subsequently, the data were smoothed with a fourth-order digital Butterworth (low-pass) filter with cutoff frequency set on 7 Hz obtained by residue analysis^46^. Finally, the coordinates 2D were transformed into 3D global coordinates using the 3D-DLT method (direct linear transformation) in Python3. The 3D reconstruction was performed with the videos of only 2 cameras, preferably the 2 lateral ones. The central camera was used for the reconstructions in the cases when the lateral ones presented error and for the annotation of the quadrants that the ball crossed the goal line.

The calibration was performed using eleven points of the soccer goal corners and the error was calculated by reconstructing the own goal. The error for each axis was: X (anteroposterior) = 0.054 m, Y (mediolateral) = 0.015 m and Z (vertical) = 0.017 m. The anatomical references obtained through OpenPose aimed the delimitation of 14 body segments: head, trunk, arms, forearms, hands, thighs, legs and feet. For this, 25 anatomical references were identified as shown in Fig. 2.

The CM calculation was performed according to the segmental method^47,48^. The volunteers’ mass and height measurements together with the 3D global anatomical references coordinate data were used to obtain the mass and CM of each body segment, using standardized anthropometric parameters^49^.

For analysis, the goalkeeper dives were synchronized and normalized in the time series (0–100%). The diving save impulse cycle was defined with its beginning when the foot ipsilateral to the diving side leaves the ground for the first time and ending at the moment the CM reaches the resulting peak velocity generated by the impulse. These references for the diving cycle were chosen so that is possible to analyze trials in which the goalkeepers defended the penalty shot or not. In this way the analyzes are not restricted to trials in which the goalkeepers touch the ball as other studies used as the ending of the diving cycle^20,21,23,43^. This diving save time series normalization is necessary for the comparison between the different dives and it also allows to infer at what moment of the diving save impulse cycle we can find differences. This technique allows a deeper understanding of the movement pattern and its effects on the velocity.

The variables chosen for our study were based on prior research involving kinematic analysis of goalkeeper diving saves^20,21,23,24,36^. Center of mass velocity and time to reach peak velocity were chosen as parameters for a comprehensive performance assessment. The other variables were chosen to quantify the goalkeepers' diving movement patterns. Each variable corresponds to an instructional tip provided in the video, so that it is possible to measure the impact of each instruction on the goalkeeper's movement. The calculation of the interested variables was performed in Python 3 as follows:

- Peak resultant velocity (PRV) = it was identified the highest value of CM velocity in the time series from the beginning of the dive until the last contact of the leg ipsilateral to the dive side with the ground plus 6 frames. The 6 frames were adopted so that there was no influence from the fall gravity acceleration in the CM velocity, which wouldn’t correspond to the impulse generated by the goalkeeper which is the study focus. The PRV was used to determine the end of the diving time series.
- Knee flexion/extension angle = it was calculated by the relative angle between the thigh and shank segments^50^, formed by the points: hip (9 or 12), knee (10 or 13) and ankle (11 or 14) (Fig. 2).
- Time to reach peak resultant velocity (TRPRV) = it was calculated through the absolute time between the moment that the lower limb ipsilateral to the dive side leaves the ground the first time and the moment the goalkeeper reaches the peak resultant velocity.
- Frontal step distance (FSD) = it was calculated only on the anteroposterior axis through the difference of the point representing the big toe (19 and 22) (Fig. 2) between the first and the last moment the ipsilateral foot leaves the ground.
- Distance between the legs in the preparatory posture (DBLPP) = for this calculation it is necessary to know the leg length of the athletes. It was calculated using the points hip (9 or 12), knee (10 or 13) and ankle (11 or 14). The points of the ankle had their vertical axis extrapolated to the heel point value (21 or 24) to represent the closest point to the ground and greater fidelity to the size of the goalkeeper's leg. Then, the distance between the heels’ points (21 and 24) was measured to infer the distance of the legs in the preparatory posture in the first moment that the foot ipsilateral to the dive side made its last contact with the ground. Finally, a relationship was performed between the legs distance in the preparatory posture and leg size and multiplied by 100 to convert to percentage.
- Frontal departure angle (FDA) = it was calculated the angle formed by the CM at the first and last moment of the ipsilateral leg contact to the ground.

A total of 154 dives were processed, and 30 were discarded, leaving 124 validated. The number of trials discarded were equal for both groups. Attempts were discarded due to the ball launch location not being ideal for the goalkeepers to perform the dives correctly. Two discarding situations prevailed: (1) when the ball was launched too close to the center of the goal; (2) when the ball was thrown too high, which modifies the goalkeeper's diving movement pattern^20^. This data cleaning was necessary so that the diving save performed by both groups were comparable. All subjects had the same number of validated trials in their pre and post situations. That is, if the participant had 4 valid trials in the pre situation for the DLL side he would have the same amount in the post situation for this same side.

### Statistical analysis

Shapiro–Wilk, Levene and Mauchly tests were performed to identify normality, homogeneity and sphericity of the data, respectively. The results were described in mean and standard deviation, the analyses were performed using a two-way ANOVA for repeated measures, and a Bonferroni post-hoc test. The independent variables were the CG and the VG and the pre and post situations, the dependent ones were the values obtained in the variables concerning the instructional video effect. Paired Student's t test was used to indicate whether there was a difference between the dives to the DLL and NDLL sides. Cohen’s d (d) was used to report the effect size of the presented variables (0.2 small, 0.5 medium, > 0.8 large). This approach not only allows for the statistical significance of findings but also provides valuable insights into the practical significance of the observed effects.

Temporal graphs were used to present the resulting velocity and the knee flexion/extension angle. Student's t test for paired samples using the method Statistical Parametric Mapping (SPM) indicated whether there were differences between the pre and post situations in both groups, between the CG and VG in both situations and in relation to the dive sides. This method of statistical analysis makes it possible to identify when in the time series the differences occur and has been used previously for biomechanical analysis^51,52^. In all cases the significance level was *p* < 0.05 and the analyzes were conducted in Python 3 and SPSS (v.21.0, IBM Statistics).

## Results

**Figure 3:**
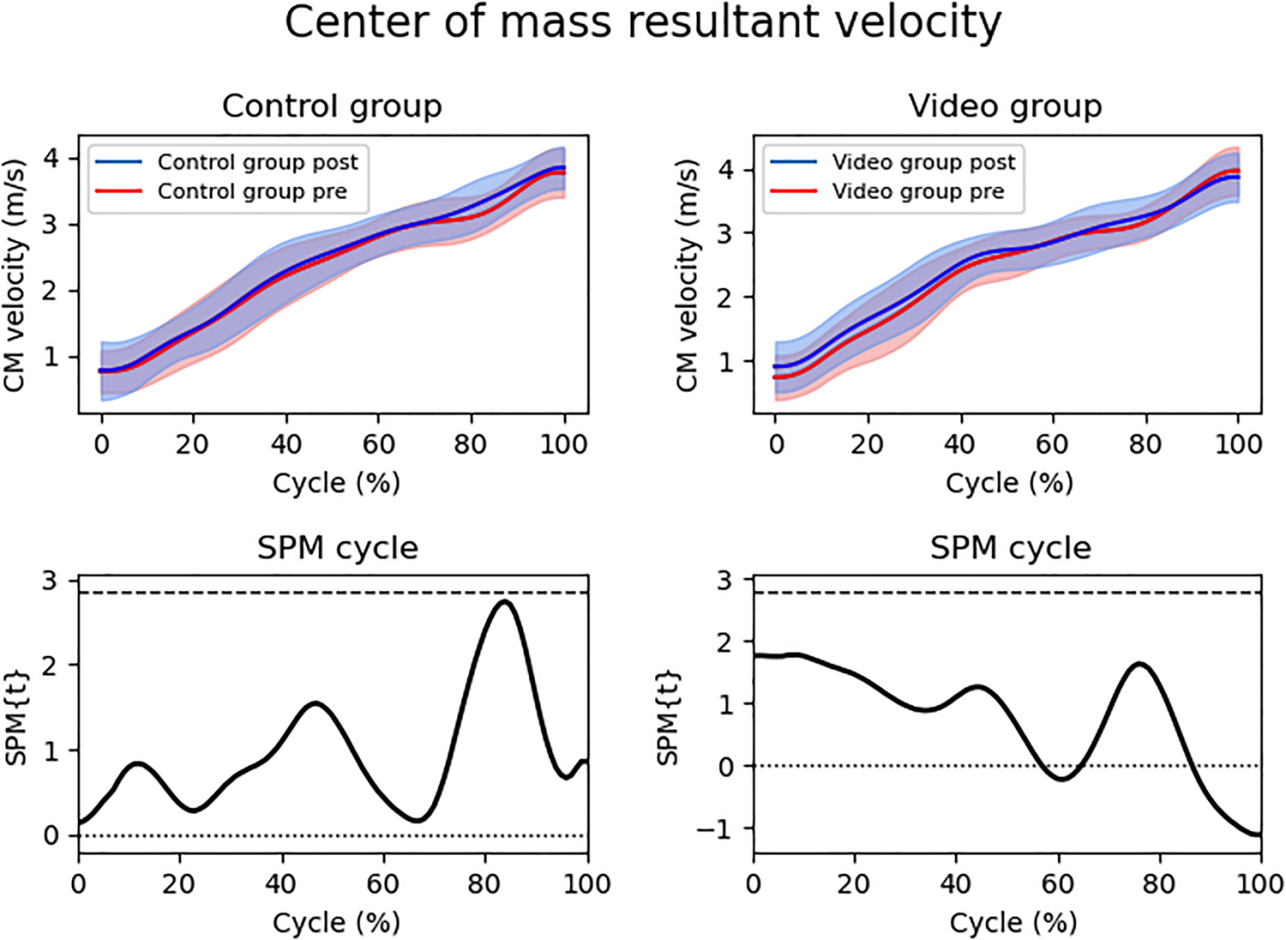
Center of mass resultant velocity between pre and post intervention situations (top) and SPM cycle (bottom) normalized in the time series (0–100%). The upper graphs represent the mean and standard deviation (shaded area) of the center of mass velocity, while the lower graphs represent the SPMt. The shaded area in the lower graphs indicates significance (*p* < 0.05). SPM = Statistical Parametric Mapping; SPM{t} = t value along the diving cycle; CM = center of mass.

No significant difference was found in the goalkeepers resulting CM velocity normalized in the time series, both in the comparison of pre and post situations of the CG (*p* > 0.05; t = 2.84) and VG (*p* > 0.05; t = 2.77) (Fig. 3) and in the comparison between CG and VG in pre (*p* > 0.05; t = 2.78) and post (*p* > 0.05; t = 2.73) situations.

**Figure 4:**
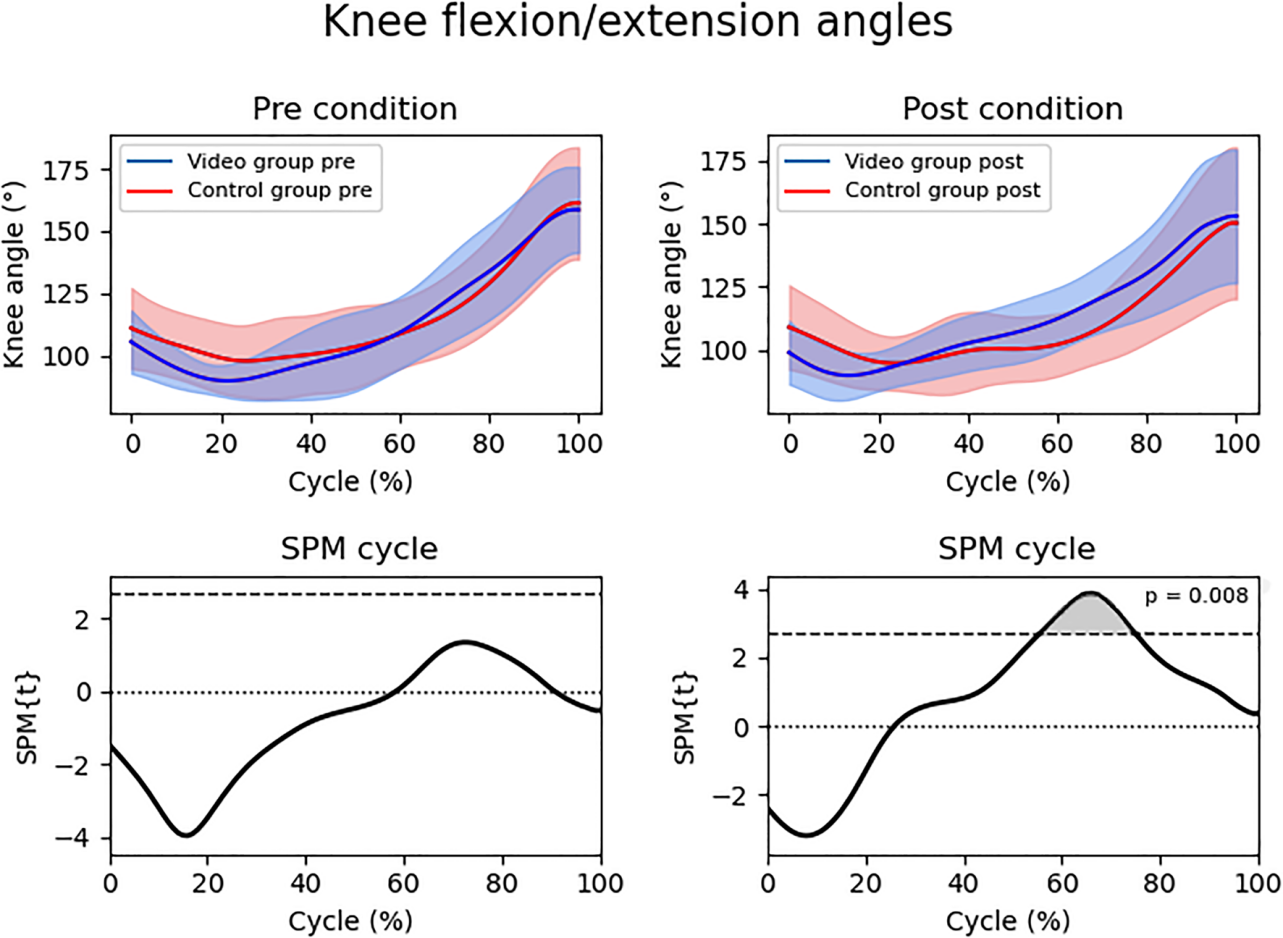
Flexion/extension angle of the knee ipsilateral to the diving side between the control and video groups in the pre and post intervention situations (top) and SPM cycle (bottom) normalized in the time series (0–100%). The upper graphs represent the mean and standard deviation (shaded area) of the knee ipsilateral to the diving side flexion/extension angle, while the lower graphs represent the SPMt. The shaded area in lower graphs indicate significance (*p* < 0.05). SPM = Statistical Parametric Mapping; SPM{t} = t value along the diving cycle.

Regarding the knee flexion/extension angle ipsilateral to the dive side, there was also no significant difference found between the pre and post situations in the CG (*p* > 0.05; t = 2.72) and VG (*p* > 0.05; t = 2.71) and in the comparison CG and VG in the pre situation (*p* > 0.05; t = 2.69). Although, the VG presented a greater knee flexion/extension angle in relation to the CG in the post intervention situation during 55.3–75% of the diving impulse cycle (*p* = 0.008; t = 2.69) (Fig. 4).

The two-way ANOVA for repeated measures showed that the VG, in the post situation, obtained higher values when compared to the CG in the same situation in the variables FSD (*p* = 0.008, d = 0.75) and FDA (*p* = 0.035, d = 0.56) and minor values in TRPRV (*p* = 0.01, d = 0.69). In the other comparisons (CGPRE x CGPOST, CGPRE x VGPRE, VGPRE x VGPOST) no significant difference was found between the variables: TRPRV, FSD, DBLPP and FDA (*p* > 0.05) (Table 1). It is worth noting that the CG's FSD in the post situation did not pass the normality test (*p* = 0.018) due to an outlier that, if replaced by the median would show normality (*p* = 0.194) and would continue to show significant difference compared to VG.

**Table 1:** Mean and standard deviation of the variables collected to verify the instructional video effect on the dives.

Variables	Control group		Video group	
	Pre	Post	Pre	Post
Time to reach the peak resultant velocity (s)	0.544 ± 0.085	0.572 ± 0.085*	0.535 ± 0.08	0.517 ± 0.075*
Frontal step distance (m)	0.313 ± 0.21	0.336 ± 0.15*^+^	0.426 ± 0.21	0.49 ± 0.247*
Distance between the legs in the preparatory posture (%ls)	0.728 ± 0.14	0.754 ± 0.144	0.784 ± 0.162	0.799 ± 0.145
Frontal departure angle (°)	13.71 ± 6.706	12.064 ± 6.102*	15.037 ± 6.973	15.708 ± 6.791*

s, seconds; m, meters; %ls, leg size percentage; °, degrees

*p < 0.05. Two-way ANOVA for repeated measures.

^+^did not pass the normality test.

**Figure 5:**
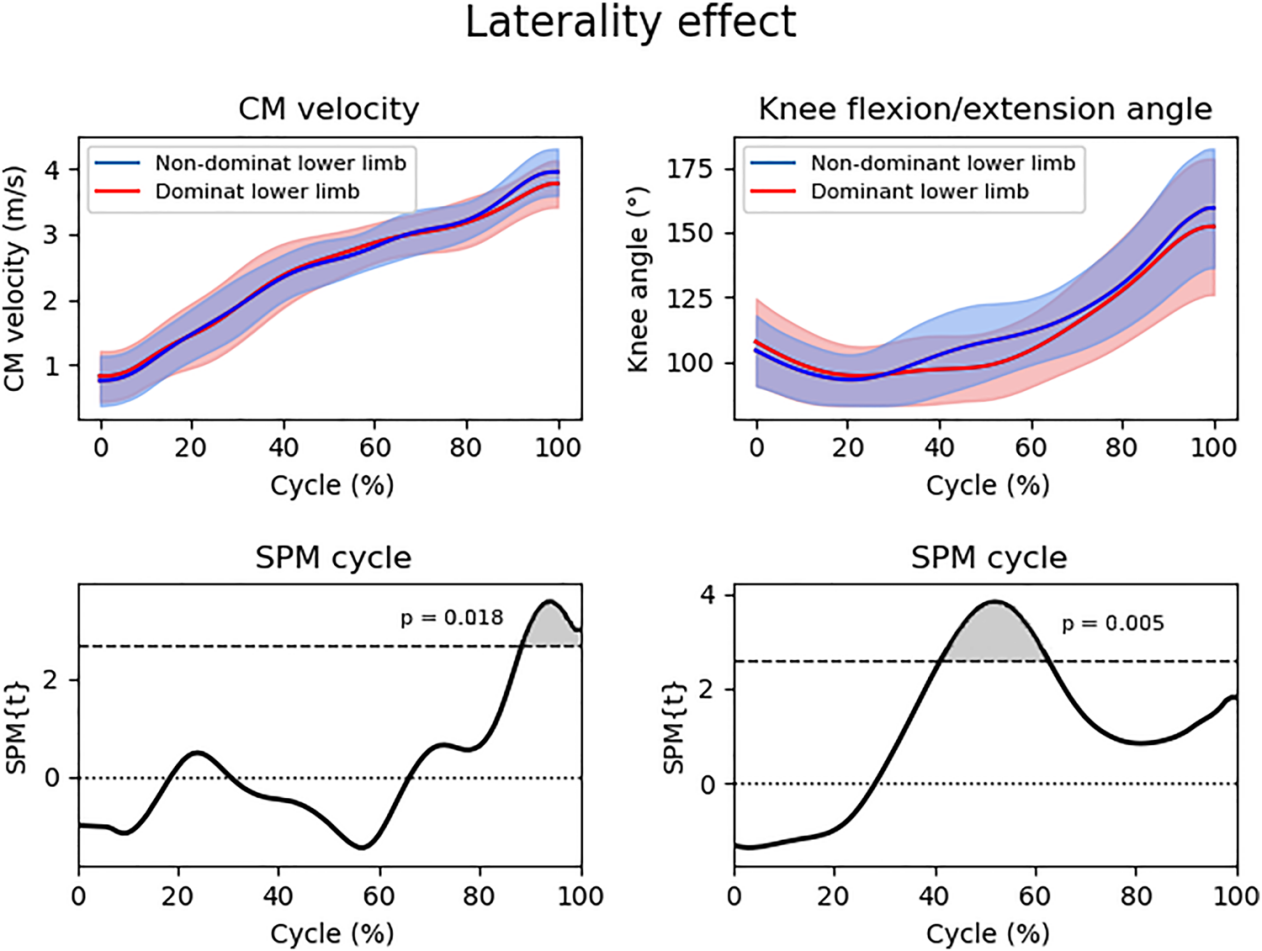
Center of mass resultant velocity and flexion/extension angle of the knee ipsilateral to the diving side between attempts to dominant and non-dominant leg side (top) and SPM cycle (bottom) normalized in the time series (0–100%). The upper graphs represent the mean and standard deviation (shaded area) of the center of mass resultant velocity and the flexion/extension angle of the knee ipsilateral to the diving side, while the lower graphs represent the SPMt. The shaded area in the lower graphs indicates significance (*p* < 0.05). SPM = Statistical Parametric Mapping; SPM{t} = t value along the diving cycle; CM = center of mass.

Regarding the laterality effect, higher values of CM velocity were found in attempts to the NDLL side during 88.4 – 100% of the diving cycle (*p* = 0.018, t = 2.68). Attempts for the NDLL side also showed greater knee flexion/extension angles during 41.3–62.6% of the diving cycle (*p* = 0.005; t = 2.61) (Fig. 5). Student's t test for paired samples indicated that the dives to the NDLL side obtained higher values in the FSD (*p* = 0.009, d = 0.45) when compared to the dives to the DLL side.

## Discussion and implication

The present study objectives were: (1) to compare the diving kinematic performance of soccer goalkeepers at the penalty moment before and after watching an instructional video that contained tips for improving the diving movement pattern, and (2) to verify the effect of laterality on this phenomenon. Although the resulting velocity did not show a significant difference between the different conditions and groups, interesting results were found regarding the instructional video effect on the VG movement pattern in relation to the CG in the post condition.

The VG presented lower TRPRV than the CG only in the post situation. Considering that there was no difference between the resulting velocity reached by the groups, it can be concluded that the VG reached the same velocity in less time, that is, it obtained a greater acceleration than the CG, which can help the goalkeepers to reach the goals corners faster and possibly increase their chance of making a defense^53^. It is worth noting that the VG had a decrease in the TRPRV from the pre to the post situation, while in the CG there was an increase. This may have been caused by fatigue and the instructional played an important role in changing the VG diving movement pattern in the post situation, making them present lower TRPRV than the CG.

The differences in the movement pattern between the CG and VG in the post situation could be noticed by observing the different values presented in the variables: angle of knee flexion/extension normalized in the time series, FSD and FDA. It is worth noting that the instructional video had tips for: (1) Use of elastic force in impulsion; (2) use the frontal step in the dive and (3) adopt a frontal departure angle as close as possible to 18°. Therefore, the instructional video acute use changed the VG goalkeepers’ movement pattern in the post situation when compared to the CG.

The difference in knee flexion/extension angle demonstrates that the VG presented greater elastic force use in the diving impulse. It can be noted by the normalized time series analysis (Fig. 4) that the VG in the post situation realized the knee flexion and extension before the CG that remained with the knee flexed for more time, taking longer to perform the extension and consequently losing part of the elastic energy of the quick movement down (knee flexion) and up (knee extension). The elastic force generated by the quick shortening-lengthening muscle cycle is an important factor for strength development^41,42^ and contributed to the VG to present lower values of TRPRV and consequently higher values of acceleration. Another instruction in the video was about DBLPP, no difference was noticed between the groups and conditions. However, it is worth noting that the instructional video tip was to the goalkeepers to spread the legs in a distance as close as possible to 75% of their leg size^43^ and they already had a preparatory posture with values ​​close to this in both the CG and VG in pre and post situations (Table 1).

Regarding the laterality effect, the present study demonstrated that the dives to the NDLL side obtained greater resultant velocity during 88.4 – 100% of the diving cycle. This happened due to the better use of elastic force when compared to the DLL side dives. It can be noticed by the similar knee flexion/extension angle behavior difference that occurred between the CG and VG in the post situation (Fig. 4). In addition, FSD may also have contributed to this higher velocity generation^24^ (Table 2).

**Table 2:** Mean and standard deviation of the variables analyzed in the dives to the side of the dominant and non-dominant lower limb.

Variables	Dominant lower limb	Non-dominant lower limb	*P* value
Time to reach the peak resultant velocity (s)	0.538 ± 0.08	0.546 ± 0.086	0.583
Frontal step distance (m)	0.343 ± 0.181	0.439 ± 0.239	0.009*
Frontal departure angle (°)	12.998 ± 6.686	15.262 ± 6.607	0.051

s, seconds; m, meters; °, degrees.

*p < 0.05. Student's paired t test.

The authors suggest that the goalkeepers that participated in this study may have a stronger DLL when compared to the NDLL and this could be another factor that helps explaining the greater velocity values in the trials to the NDLL side. That would happen because the lower limb contralateral to the dive side contributes more than the ipsilateral to the velocity obtained by the CM^20^. The asymmetry tending to a better performance to the NDLL side was also found in other study, but only in the diving save displacement^24^. The asymmetry in the velocity differs from other studies that found greater values in the dives to the DLL side^23^ and no differences between the velocities obtained in the dives to both sides^20,25^. However, it is important to highlight that all these studies were carried out in a laboratory and with stationary balls, while the present study was carried out in the field with balls in movement.

The primary limitation of the present study was related to the condition of the ball launching machine on the data collection day. Due to its suboptimal functioning, some balls were thrown in a manner that adversely affected the goalkeepers' dives, leading to the exclusion of 30 trials. It's important to note that, like any mechanical and electrical equipment, the ball launcher's performance was susceptible to the high ambient temperature which can cause operational variations. This is an inherent and normal challenge in conducting scientific tests under real-world conditions. Furthermore, being on a natural grass field, the machine often altered the turf's surface, affecting the vertical trajectory of the ball launch. To mitigate this, the machine was recalibrated as necessary. While these factors contributed to the exclusion of several attempts, they also reflect the realities of data collection in an ecologically valid setting, which is a crucial aspect of conducting field-based research.

In addition, in the current study only the acute effect of the instructional video was tested, future researches should be done to verify if there is retention of the new movement pattern performed by the athletes. The low number of participants is also a limitation, but it is in accordance with other goalkeeper diving save studies because of the comparatively lower number of athletes playing in this position^20–25,36,43,53^. The main limitation with OpenPose was the necessity for manual corrections. This requirement for manual intervention highlights a broader challenge within the field of markerless motion analysis^45^. For markerless methods to be more widely adopted in sports contexts, there is a need for advancements in the precision of body segment identification to reduce or eliminate manual interventions, enhancing usability and accelerating adoption in sports research and practice. The authors believe that the current study opens doors for future researches because it demonstrated that it is possible to: (1) perform kinematic analysis without the need for attached markers to the athletes' body, (2) carry out all the collection in the field for greater ecological validity and (3) use balls in movement for kinematic analysis of goalkeepers diving saves.

Despite the study limitations, the proposed methodology for in field kinematics analysis can be an important tool for coaches to understand their goalkeepers’ diving save, monitoring the performance and indicating the athletes’ advantages, deficiencies, and limitations in their diving movement pattern. Furthermore, the video instruction can be an important tool to make refinements in specialized and experienced goalkeepers movement pattern and be used alongside with strength and power training methods based on the recent findings in goalkeeper diving save kinetics and kinematics to improve the athletes’ performance^22^. The diving acceleration improvement can give the goalkeepers more time to decide which side to dive, permitting them to collect more information about the penalty taker kicking movement technique improving their chance to anticipate correctly^54–58^. All of this can contribute to increase the success rate in penalty save attempts.

## Conclusion

This study demonstrated that the instructional video was effective in generating an acute change in the diving movement pattern of specialized young goalkeepers in the groups’ comparison. In the post situation the video instruction group showed a difference concerning the control group in the variables: knee flexion/extension angle, time to reach peak resultant velocity, frontal step distance and frontal departure angle which generated a greater acceleration in the dive. In relation to laterality, it was found that attempts to the non-dominant lower limb side showed greater resultant velocity during 88.4–100% of the diving cycle, different patterns in the knee flexion/extension angle and higher values in the frontal step distance.

## Data Availability

The datasets generated in the current study, the code for the data analysis and the instructional video, the original and with English subtitles, are available on: https://github.com/rafaellmmonteiro/FAPESP_2020-14845-6-Instructional_video_goalkeeper and https://doi.org/10.6084/m9.figshare.23507793 (As on april. 2023).

## Abbreviations

CG: Control group
VG: Video group
CM: Center of mass
NDLL: Non-dominant lower limb
DLL: Dominant lower limb
IPLAG: Global lateral preference inventory
VGPRE: Video group in the first 10 trials
VGPOST: Video group in the last 10 trials
CGPRE: Control group in the first 10 trials
CGPOST: Control group in the last 10 trials
PRV: Peak resultant velocity
TRPRV: Time to reach peak resultant velocity
FSD: Frontal step distance
DBLPP: Distance between the legs in the preparatory posture
FDA: Frontal departure angle
SPM: Statistical parametric mapping
